# Voyage of discovery? A comment on Koch et al. “A voyage to Terra Australis: human-mediated dispersal of cats”

**DOI:** 10.1186/s12862-016-0813-y

**Published:** 2016-12-07

**Authors:** Rose L. Andrew, Deane Smith, Jamieson C. Gorrell, Jasmine K. Janes

**Affiliations:** School of Environmental and Rural Science, The University of New England, Armidale, NSW 2351 Australia

**Keywords:** Feral cat, Migrate-N, Structure, Microsatellite, Migration matrix

## Abstract

The origins of feral cats in Australia may be understood with the help of molecular studies, but it is important that hypotheses be tested with appropriate sampling and methodology. We point out several shortcomings in the analysis by Koch et al. (BMC Evol Biol 15:262, 2015; A voyage to Terra Australis: human-mediated dispersal of cats. Dryad Digital Repository, 2015), present a reanalysis of part of the study and discuss the challenges of elucidating the early history of feral cats.

## Main text

In a recent issue of this journal, Koch et al. [1] presented the first molecular study addressing a compelling evolutionary question – the origins of feral cats in Australia. Feral populations of domestic cats (*Felis catus*) are a serious problem in Australian conservation [2–4]. The intriguing possibility that cats were introduced to Australia from Asia prior to European settlement [1, 5], if true, would potentially decouple the decline and extinction of many native mammals from the rise of cats. Evidence from historical records contradicts this hypothesis, but does not offer definitive proof [6, 7]. While genetic data could potentially hold the key to this problem, such a study must be based on sound and appropriate techniques. We have identified two principal deficiencies in the paper by Koch et al. [1]. We describe both below, with support from a reanalysis of part of the study, and discuss the difficulty of firmly ruling out a pre-European introduction of feral cats using molecular data.

### Identifying colonisation pathways

While we agree that a substantial portion of the early Australian feral cat population was probably supplied by Europeans, the model selection approach used Koch et al. [1] was not appropriate to the task of elucidating the origins of the population. The first hypothesis that they aimed to test was whether cats arrived from Asia prior to European settlement. This is a question that the software used, Migrate-N [8], appears unsuited for. Migrate-N fits an island model at migration-drift equilibrium [8, 9], making it suitable for studying patterns of ongoing, stable migration. The appropriate use of Migrate-N in phylogeography is to estimate migration rates and effective population sizes simultaneously, for example in the case of asymmetric migration rates between cave and surface populations in the Mexican blind cavefish complex [10]. It is not designed to distinguish between recent gene flow and historical colonisation [8, 9], or to identify primary and secondary invasion routes, as Koch et al. [1] have interpreted their results.

Even if the research question concerned migration rates alone, key assumptions of Migrate-N are likely to be broken in Australian feral cats. First, migration-drift equilibrium appears unlikely in this system: regardless of whether a population of Asian origin already existed in Australia, the bulk of the European contribution is likely to have occurred very recently (i.e. in the 228 years since British colonisation). Although the population may have been relatively stable, the rates of gene flow into the population are unlikely to have remained constant during that interval (e.g. recent trends in desexing; [11, 12]). Second, the dataset is missing populations of stray and domestic cats in Australia, as well as populations from Asian locations other than Malaysia and Sulawesi. A “ghost” population should be considered in the analysis to satisfy the assumption that all populations have been sampled [13]. Finally, each population is assumed to be panmictic, which more careful analysis of the microsatellite data would have shown to be false (see below). The lack of estimated migration matrices or probability intervals made it difficult to assess whether a realistic final model was obtained for feral cats [1].

Migrate-N is unable to determine whether ancestral polymorphism or gene flow is responsible for shared polymorphism among Europe, Asia and Australia, but more suitable coalescent-based methods for modelling isolation with migration and estimating divergence times require multiple loci, and some of the more popular methods (e.g. IMa2 or LAMARC; [14, 15]) also require the population tree to be specified a priori. More flexible Approximate Bayesian Computation approaches (e.g. PopABC, [16]) are likely to be needed to distinguish between the complex sets of possible demographic histories that could have produced Australia’s current feral cat population [17].

The interpretation of the mitochondrial phylogeny was more appropriate, despite the lack of intraspecific differentiation in the cat [18]. Although it is surprising that only a Bayesian tree was shown, without corroboration from other approaches such as maximum likelihood (ML), there is evidence in the phylogeny presented by Koch et al. [1] of lineage sorting in some populations; only Subclade A was found on the Cocos (Keeling) Islands, Dirk Hartog Island, Tasman Island and Flinders Island, as well as in several Western Australian populations (although the sample size was rather low in the Tasman Island and Flinders Island populations). The links to historical human movements highlighted by Koch et al. [1] support their suggestion that the island populations reflect the genetic composition of early cat introductions, and perhaps the same applies to the Western Australian populations.

However, results based on organelle markers, which represent a single linked locus, should be interpreted cautiously, and several important parameter choices, such as substitution model and tree prior, appear to be missing from the methods section [1]. Furthermore, the occurrence of both major mitochondrial subclades in both Asian and European populations in approximately equal frequencies implies little power to distinguish relationships with the Australian feral cat populations. Indeed, both subclades also occur in the wild relative of *F. catus*, *F. silvestris lybica* (Near Eastern Wildcat), and subclade A even occurs in the Central Asian Wildcat, *F. s. ornata* [18]. There is little resolution in the tree, but the few supported (HPD > 0.7) clades with > =3 samples include either a single geographic region or representatives of all three regions of interest (mainland/Tasmania, Europe and Asia). As a result, there is little power to infer population relationships. The “secondary introductions” favoured by Koch et al. [1] cannot be inferred from either the Migrate-N analysis or the mitochondrial phylogeny. An alternative explanation for the observed topology, suggested by a thoughtful reviewer, is that the Asian cats sampled from Singapore and Malaysia have European ancestry, resulting from the immigration of European cats documented in earlier work [18, 19]. Nuclear data are needed to distinguish between European and Asian random bred cats, however, as no phylogeographic association has been observed among mitochondrial subclades [18]. Without more Asian samples in the nuclear DNA analysis, it is impossible to verify whether these cats represent Asian heritage.

### Genetic structure of Australian feral cat populations

Koch et al. [1] used the program STRUCTURE [20, 21] to study the genetic structure of 265 cats samples across mainland Australia and the surrounding islands (262 cats), as well as Malaysia (three cats). The lack of Asian samples in the nuclear data set prevented its use in testing the hypothesis of an Asian origin, which another recent study was able to do [5]. Instead, the key findings reported were a lack of structure within mainland Australia and high differentiation between mainland Australia and the offshore islands. The Malaysian samples clustered with the Australian mainland group and the Cocos (Keeling) Island population clustered with Tasman and Flinders Islands. The key flaws of the analysis were insufficient MCMC run length and number of runs, which did not follow recommendations of the program manual or those of a study of the reproducibility of STRUCTURE results [22]. Koch et al. [1] used 50,000 burn-in iterations, followed by the collection of data from 100,000 iterations, and supporting information suggested that convergence had not occurred in all runs.

**Fig. 1:**
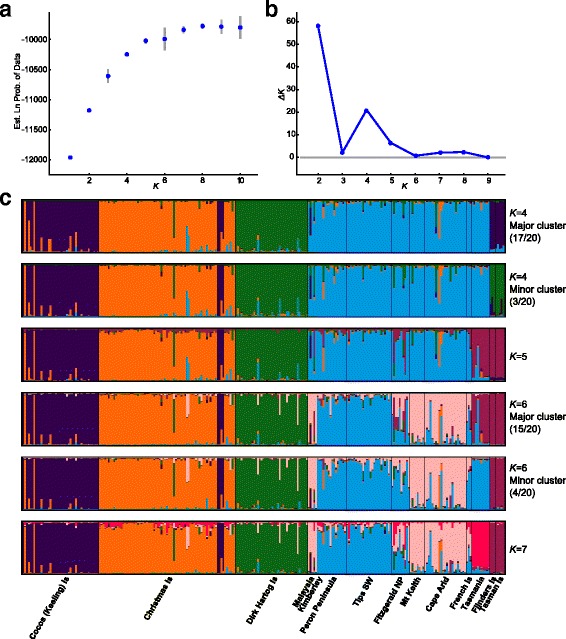
Log-likelihood (**a**) and *ΔK* (**b**) plots for reanalysis of all microsatellite data in Koch et al. [1], with 20 replicate runs of 500 000 burn-in iterations and 1 000 000 further iterations, summarised by Structure Harvester [31]. Below are ancestry coefficients (**c**) for major and minor modes (*K* = 4 to *K* = 7) identified and plotted by CLUMPAK [24, 32]

With longer runs and more replication, we were unable to reproduce either the number of populations (*K*) in the overall data set or the lack of structure within the Australian mainland using the data downloaded from Dryad [23]. Using the same model as Koch et al. [1], but with a burn-in of 500,000 iterations and a subsequent run length of 1,000,000, we found that the model specifying *K* = 5 outperformed that assuming *K* = 4 (Fig. 1). Among the 20 runs at each value of K (1 to 10), CLUMPAK [24] detected a single grouping pattern (“mode”) at values of 1–3, 5 and 7. In contrast, the value favoured by Koch et al. [1], *K* = 4, led to the offshore Tasmanian islands (Tasman and Flinders Islands) clustering with Dirk Hartog Island, rather than the Cocos (Keeling) Islands in three out of 20 runs (Fig. 1). Our reanalysis shows that these islands formed a stable grouping with Tasmania at *K* = 5, and at *K* = 7 there was evidence of a distinction between the Tasmania population and the two islands (in agreement with the PCA presented by Koch et al. [1]). The reanalysis also provided evidence of some clustering within the Australian mainland samples, as detected by Spencer et al. [5].

**Fig. 2:**
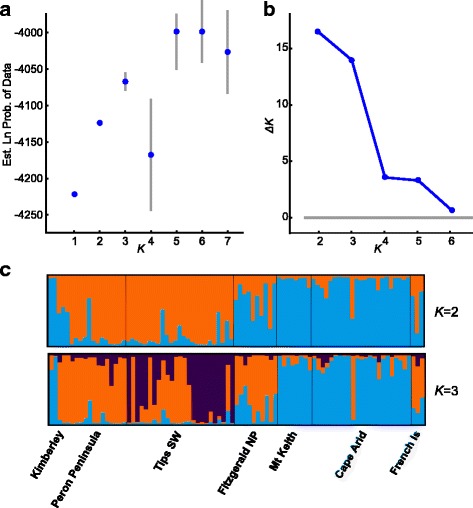
Log-likelihood (**a**) and *ΔK* (**b**) plots for reanalysis of microsatellite data from populations belonging to the major cluster for *K* = 5 in Fig. 1 (omitting Malaysia), with 20 replicate runs of 500 000 burn-in iterations and 1 000 000 further iterations, summarised by Structure Harvester [31]. Below are ancestry coefficients (**c**) for *K* = 2 and *K* = 3, averaged across runs and plotted by CLUMPAK [24, 32]

Koch et al. [1] employed the Δ*K* [25] statistic to identify the value of *K* best supported by the data, but did not follow the authors’ recommended procedure for detecting substructure. Evanno et al. [25] noted that their method does not perform well in the presence of hierarchical structure and suggested repeated rounds of analysis within the clusters detected at the optimal *K*, in order to detect further substructure. We favour the approach of Pritchard et al. [20], which also recommends reanalysing clusters that may harbour substructure. If Koch et al. [1] had examined substructure, they would have most likely detected groupings within the feral cats of mainland Australia (shown in red on their Fig. 2, [1]). Our reanalysis of the largest cluster in the global analysis (at *K* = 5) detected three groups (Fig. 2), corresponding to the “pan-Australian” and “coastal Western Australian” clusters identified by Spencer et al. [5], as well as a third cluster occurring in rubbish dump populations in southwestern Western Australia. Approximately half the cats of such provenance were estimated to have ~90 % ancestry belonging to the third cluster, suggesting that the third group is not an artefact. Malaysia was omitted from this analysis, as the sample size was extremely small (three animals).

In summary, we disagree with two of the main conclusions of the microsatellite analysis of Koch et al. [1]: genetic structure within the Australian mainland populations is detectable with this data set and there is serious doubt as to whether the Tasman Island and Flinders Island populations cluster with the Cocos (Keeling) Islands.

### Elucidating the history of feral cats

Understanding the contributions of different sources of introductions to feral cat populations in Australia is interesting from an evolutionary perspective, as it could hold the key to differences in morphology and behaviour among bioregions. However, we must not jump to conclusions based on flawed analyses of a small dataset. Even careful analysis of a large microsatellite dataset [5] provides a somewhat simplified picture of what is likely to be a complex history. In our opinion, resolution of the history of the feral cat in Australia will probably require a powerful multilocus marker set, as well as improved sampling of potential source populations and domestic breeds whose genetic variation may have been added to the feral gene pool [26].

The difficulty of trying to detect pre-European introductions is the large amount of gene flow that has probably occurred in the intervening years from domestic cats of European origin. It is possible that only a small fraction of Asian ancestry would persist, so any analysis would have to be extremely powerful to effectively rule out an Asian contribution. Larger marker sets would be needed to confidently exclude an Asian origin, but even they might fail to detect a small amount of Asian ancestry without a suitable reference population of Asian cats. Genome-wide marker data would also enable testing of whether Asian ancestry persisted in the Australian feral cat population partly as a result of natural selection [27].

We hope to have shed some light on the structure that can be detected with microsatellites, as well as the reasons that more appropriate methods and data are needed to understand what we can of the history of feral cats in Australia.

## Funding

This work did not require external funding.

## Authors’ contributions

RA, JG and JK planned the response, RA wrote the first draft of the manuscript, DS conducted the STRUCTURE reanalysis, and all authors edited the manuscript.

## Acknowledgements

We thank Fran Zewe and Guy Ballard for initial discussions of the study.

## Ethics approvals and consent to participate

Not applicable.

## Consent to publish

Not applicable.

## Availability of data and material

All data used in this correspondence article is found in the original article and its supporting data [1, 23].

## Abbreviation

*K*: The number of populations assumed or estimated in analysis of genetic data by the program STRUCTURE
