# Supplementary material for: Voyage of discovery? A comment on Koch et al. “A voyage to Terra Australis: human-mediated dispersal of cats”
Source: BMC Evol Biol. 2016 Dec 7;16:270. doi: 10.1186/s12862-016-0813-y (PMC5142327; doi:10.1186/s12862-016-0813-y)
Supplement: Related texts

# Reply: Response

**Authors:** K. Koch, D. Algar, J. B. Searle, M. Pfenninger, K. Schwenk

**Affiliations:** 2 Biodiversity and Climate Research Centre (BiK-F) by Senckenberg Naturforschende Gesellschaft and Goethe-University, Senckenberganlage 25, 60325 Frankfurt am Main, Germany; 3 Department of Parks and Wildlife, Science and Conservation Division, Senckenberganlage 25, 60325 Frankfurt am Main, Germany; 4 Department of Parks and Wildlife, Science and Conservation Division, Senckenberganlage 25, 60325 Frankfurt am Main, Germany; 5 Department of Parks and Wildlife, Science and Conservation Division, Senckenberganlage 25, 60325 Frankfurt am Main, Germany

The authors thank Andrew et al. for their Correspondence and their additional interpretations with regard to the human-mediated dispersal of cats [1]. However, their criticism is misaddressed at least in part, and the suggested extra analyses do not provide substantial new information.

Andrew et al. identified two aspects of concern, the first one refers to the fact that we used a model selection approach (based on Migrate-N) to answer the question whether cats from Asia arrived prior to European settlements. We fully agree with Andrew et al. that this approach is not designed to study temporal aspects of migration routes. However, neither in the Introduction, nor in the Discussion, have we claimed that we intended to differentiate among current and past gene flow. Instead we studied the structure of gene flow routes. Furthermore, we based our overall interpretation of the invasion history of cats on a variety of analyses and datasets (e.g. phylogenetic tree, migration model selection approach, microsatellite data and analyses, and historical data). Any interpretation related to samples with a low number of individuals (e.g. Asia) have been adequately discussed.

The second aspect of concern is related to the analysis of DNA microsatellite data using the program STRUCTURE [20]. We agree with Andrew et al. that longer runs and more replication and most importantly adding a reanalysis using only a fraction of the samples (one cluster at *K* = 5) does result in an additional substructure (three groups instead of one). The outcome of this analysis, however, does not alter the main conclusion of the manuscript, rather adds a minor aspect to the overall picture. In addition, if such an ‘hierarchical STRUCTURE analysis’ is applied then q thresholds as well as alternative methods (e.g. BAPS) are required (see e.g. [28]).

In summary, we acknowledge that in addition to our results on the voyage of cats to Terra Australis [1, 29, 30] there may be differentiation that we did not detect among Australian mainland feral cat populations and that feral cat populations from Tasman and Flinders island might show differences from those of Cocos (Keeling) islands that we did not detect.
